# Multiplex analysis of cytokines in the cerebrospinal fluid of dogs after ischemic stroke reveals elevations in chemokines CXCL1 and MCP-1

**DOI:** 10.3389/fvets.2023.1169617

**Published:** 2023-05-17

**Authors:** Renee M. Barber, Simon R. Platt, Luisa De Risio, Jamie Barber, Kelsey R. Robinson

**Affiliations:** ^1^Department of Small Animal Medicine and Surgery, University of Georgia College of Veterinary Medicine, Athens, GA, United States; ^2^Linnaeus Veterinary Limited, Shirley, United Kingdom; ^3^Department of Infectious Diseases, University of Georgia College of Veterinary Medicine, Athens, GA, United States

**Keywords:** dog, stroke, cerebrovascular accident, cerebrospinal fluid, cytokine, chemokine, CXCL1, MCP-1

## Abstract

**Introduction:**

Neuroinflammation that occurs in the brain after stroke has been shown to be important to disease pathogenesis and outcomes. The aim of this study was to evaluate a large number of pro- and anti-inflammatory cytokines in dogs with clinically-confirmed, naturally occurring stroke.

**Materials and methods:**

Fifteen dogs with a clinical diagnosis of ischemic stroke and ten healthy control dogs were included in the study. A multiplex immunoassay was utilized to evaluate cerebrospinal fluid for GM-CSF, IFN-γ, IL-2, IL-4, IL-6, IL-7, IL-8, IL-10, IL-15, IL-18, IP-10, CXCL1, MCP-1, and TNF-α.

**Results:**

Mean concentrations of CXCL1 (stroke-436 pg/ml, control-267 pg/ml, *p* = 0.01) and MCP-1 (stroke-196 pg/ml, control-66 pg/ml, *p* ≤ 0.0001) were significantly elevated in dogs with stroke when compared with control dogs. Location and type of infarct, duration of clinical signs, and use of anti-inflammatory medications were not associated with differences in cytokine concentration.

**Discussion:**

CXCL1 and MCP-1 may play a role in naturally occurring canine stroke and represent targets for future research.

## 1. Introduction

Stroke is a common cause of acute neurological dysfunction in dogs. Although recovery with supportive care is typical, up to 23% of dogs presented for stroke do not survive (1, 2). Currently, there are no directed treatments to improve the prognosis of stroke in these patients.

In other species, the neuroinflammation that occurs after acute ischemic stroke has been shown to be important to disease pathogenesis. Elevations of TNF-α, IL-1, and IL-6 are associated with larger infarct zones, and their reduction ameliorates clinical disease in animal models. All three of these cytokines have been detected in the cerebrospinal fluid (CSF) of people after stroke, and elevations of TNF-α in CSF have been associated with poor clinical outcomes (3).

Understanding the CNS inflammatory response to stroke in dogs could enhance our ability to predict patient outcomes as well as the development of more effective therapeutic approaches. In canine models of stroke, elevated IL-6 in CSF has been shown to be associated with a poorer prognosis, and, more recently, IL-6 has been shown to be elevated in the CSF and plasma of dogs with naturally occurring stroke (4). To expand on this, we employed a multiplex assay to compare levels of 14 pro- and anti-inflammatory cytokines in the CSF of dogs with clinically confirmed, naturally occurring ischemic stroke to those of normal dogs. This is the first study to evaluate a large array of cytokines in the cerebrospinal fluid of dogs with naturally occurring stroke.

## 2. Materials and methods

### 2.1. Case selection

**Figure 1:**
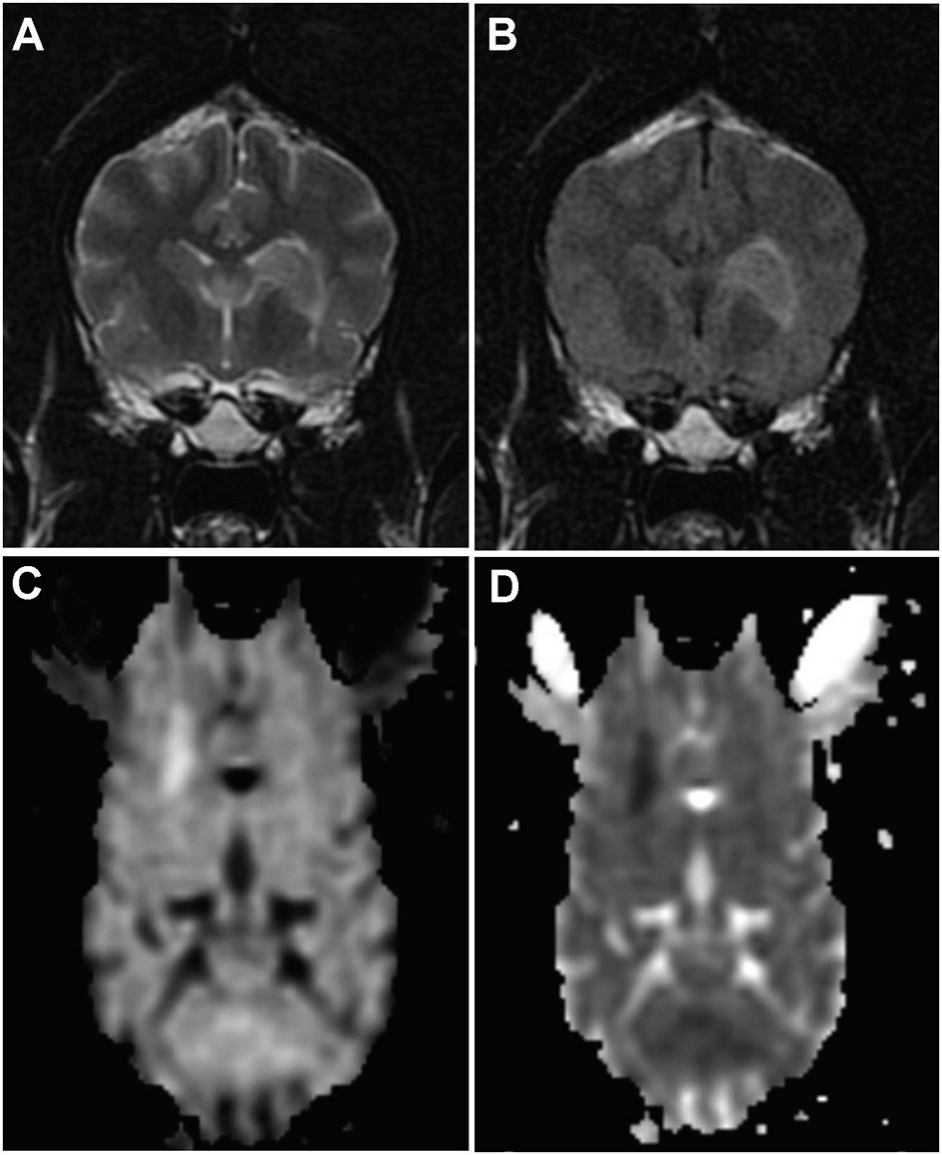
MR images from dog 10 with a presumed ischemic infarct in the vascular territory of the striate arteries on the left. Transverse T2-weighted **(A)** and fluid-attenuated inversion recovery images **(B)** of the brain show a well-defined hyperintesity in the area of the head of the caudate nucleus extending ventrolaterally to the adjacent internal capsule. The lesion is hyperintense on diffusion weighted images **(C)** and hypointense on apparent diffusion coefficient map **(D)**, consistent with restricted diffusion.

Clinical data were collected from medical records retrospectively. Patients that were presented to a specialist neurology practice at the Animal Health Trust or University of Georgia with clinical signs, magnetic resonance imaging (MRI), and CSF findings consistent with ischemic stroke were included (1, 2, 5–7). Clinical signs had to have a peracute onset with no evidence of progression. A neurological exam had to reveal a focal, lateralizing prosencephalon or cerebellar anatomic localization. MRI had to be performed within 5 days of the onset of clinical signs. MRI lesions had to be well demarcated with hyperintensity on T2-weighted and/or fluid-attenuated inversion recovery sequences that was predominantly confined to gray matter, with minimal to no mass effect, and consistent with a vascular lesion distribution; DWI had to be hyperintense and ADC had to be hypointense, which is restricted diffusion (Figure 1). Medical records and imaging were utilized to record signalment, location, and type (territorial vs. lacunar) of stroke; (7) findings of CSF analysis; time of disease onset prior to sample collection; and prior use of anti-inflammatory medications.

Fifteen dogs with a clinical diagnosis of ischemic stroke were identified and included in the study (Table 1). The mean age was 7.7 years (range 4–14 years). There were four males, six neutered males, and five spayed females. Breeds included Cavalier King Charles Spaniel (*n* = 2), Labrador Retriever (*n* = 2), Weimaraner (*n* = 2), mixed breed (*n* = 2), and one Border Collie, Chow Chow, Belgian Malinois, Japanese Akita, Siberian Husky, Shih Tzu, and West Highland White Terrier, respectively. Ten dogs had a territorial ischemic lesion, and five dogs had a lacunar ischemic lesion. No dogs had reported concurrent disease (e.g., cardiovascular disease, cancer). CSF analysis was considered normal in all dogs with total nucleated cell counts of <5/μl and total protein of <35 mg/dl. The duration of clinical signs prior to the onset of CSF collection ranged from 0 to 5 days (median 1 day). To determine whether the duration of signs altered cytokine detection, cases of stroke were divided into two categories: (1) onset of stroke < 48 h before CSF collection (*n* = 8) vs. ≥ 48 h before CSF collection (*n* = 7). Nine dogs did not receive anti-inflammatory medication; six dogs received various anti-inflammatory medications within 2 weeks of diagnosis and CSF collection.

**Table 1:** Descriptive data of dogs with naturally occurring stroke.

**Case**	**Breed**	**Sex**	**Age at onset of disease (years)**	**Duration of clinical signs prior to CSF collection (days)**	**MRI findings**
1	West Highland White Terrier	MN	4	0	Thalamic, lacunar ischemic infarct
2	Mixed breed	FS	8	5	Rostral cerebellar territorial ischemic infarct
3	Greyhound	M	4	0	Rostral cerebellar territorial ischemic infarct
4	Mixed breed	MN	11	0	Rostral cerebellar territorial ischemic infarct
5	Shih Tzu	MN	12	1	Rostral cerebellar territorial ischemic infarct
6	Labrador Retriever	M	9	0	Midbrain lacunar ischemic infarct
7	Japanese Akita	FS	10	3	Thalamic lacunar ischemic infarct
8	Weimeraner	MN	9	0	Thalamic lacunar ischemic infarct
9	Weimeraner	MN	9	5	Rostral cerebellar territorial ischemic infarct
10	Belgian Malinois	M	6	0	Thalamic lacunar ischemic infarct
11	Chow Chow	FS	14	1	Rostral cerebellar territorial ischemic infarct
12	Border Collie	FS	13	3	Rostral cerebellar territorial ischemic infarct
13	Miniature Poodle	MN	9	5	Rostral cerebellar territorial ischemic infarct
14	Labrador Retriever	M	11	3	Rostral cerebellar territorial ischemic infarct
15	Siberian Husky	FS	9	5	Rostral cerebellar territorial ischemic infarct

MN, neutered male; FS, spayed female; M, intact male; kg, kilogram; M, male intact; MRI, Magnetic resonance imaging.

### 2.2. Sample collection

CSF was collected from the atlantooccipital or lumbar region as part of routine diagnostic investigation from dogs presented for neurological evaluation. All CSF was evaluated by a board-certified clinical pathologist to determine cell count, protein concentration, and cytological characteristics. Excess CSF was immediately frozen and stored at −80°C for future diagnostic testing. Once CSF was no longer needed for diagnostic evaluation and was to be discarded, it was utilized for this study. Written client consent was obtained to utilize these samples for future research publications.

CSF was also collected from ten control dogs. All control dogs were healthy, 2-year-old beagle dogs that had a normal neurological exam, normal brain MRI, and normal CSF cell count and cytology at the time of sample collection. All procedures for these control dogs were approved by the University of Georgia Institutional Animal Care and Use Committee (UGA IACUC) and dogs were managed in accordance with UGA IACUC welfare protocols (protocol # A200910-058).

### 2.3. Cytokine evaluation

Prior to cytokine evaluation, CSF was centrifuged at 10,000 x g for 5 min. All CSF samples were evaluated simultaneously for GM-CSF, IFN-γ, IL-2, IL-4, IL-6, IL-7, IL-8, IL-10, IL-15, IL-18, IP-10, CXCL1, MCP-1, and TNF-α using a commercially available canine multiplex immunoassay (Milliplex MAP canine cytokine/chemokine assay, Millipore, Billerica, MA, USA) according to the manufacturer's instructions. Briefly, antibody-coated detection beads were incubated overnight at 4°C with appropriate standards, samples, or quality controls in 96-well filter plates. All wells were then incubated with biotinylated secondary detection antibodies followed by streptavidin-phycoerythrin. After the final wash, samples were resuspended in 150 μl phosphate buffered saline and read immediately on a Luminex 200 instrument (Luminex, Austin, TX, USA). The kit provided recombinant cytokines that were utilized as standards and considered positive controls for the procedure. All standards, samples, and controls were run in duplicate. Mean fluorescence intensities were analyzed using Milliplex Analyst software (Millipore, Billerica, MA) to determine concentration values. All standards and controls were within the expected concentration ranges.

### 2.4. Statistical analysis

A student's *t*-test was used to test for differences in measurements between binary factors: clinical dogs vs. normal; location of stroke (cerebrum, thalamus, cerebellum); territorial vs. lacunar infarct; duration of clinical signs (≤48 h vs. >48 h); anti-inflammatory medications administered (within 2 weeks of CSF collection) vs. none given. Q-Q plots of the residuals (using both raw and logarithmically transformed data) were examined to confirm normal data distribution. A test for equality of variances was performed. Where variances of the groups were found to be significantly unequal, the Satterthwaithe test was used to test for differences between groups. Student's *t*-tests and Satterthwaithe tests were implemented in PROC TTEST in SAS V 9.2 (SAS, Cary, NC). Significance was set at *p* ≤ 0.05.

## 3. Results

GM-CSF, IL-15, IL-18, and IP-10 were not detected in any cases or controls. IFN-γ was detected in 13/15 cases and 7/10 controls. IL-2 was detected in 10/15 cases and 10/10 controls. IL-4 was detected in 7/15 cases and 5/10 controls. IL-6 was detected in 2/15 cases and 1/10 controls. IL-7 was detected in 3/15 cases and 5/10 controls. IL-8 was detected in 1/15 cases and no controls. IL-10 was detected in 1/15 cases and 2/10 controls. TNF-α was detected in 12/15 cases and 9/10 controls (Table 2). There was no statistical difference in concentration between cases and controls or for other variables, such as type of infarct or duration of clinical signs for the above cytokines.

**Table 2:** Cerebrospinal fluid cytokine concentrations from dogs with naturally occurring stroke.

**Cerebrospinal fluid cytokine concentrations (pg/ml)**										
**Case**	**IFN-**γ	**IL-2**	**IL-4**	**IL-6**	**IL-7**	**IL-8**	**IL-10**	**TNF-**α	**CXCL1**	**MCP-1**
1	0.38	0	0	0	0	0	2.0	0.7	465.5	277.7
2	0	0	0	34.7	0	0	0	0	157.3	83.1
3	0	0	0	0	0	0	0	0	241.5	223.8
4	0.64	0	0	0	0	0	0	0	214.2	183.7
5	0.46	2.2	0	0	0	0	0	1.7	625.2	221.0
6	0.64	11.0	0	0	0	18.7	0	1.8	788.1	163.4
7	0.31	8.2	20.37	0	1.2	0	0	1.5	343.9	192.7
8	0.31	2.7	0	0	0	0	0	1.9	475.1	198.5
9	0.31	0	0	0	0	0	0	1.6	319.5	150.2
10	0.46	4.4	57.0	0	0	0	0	1.3	680.5	233.0
11	0.64	3.2	9.5	0	0	0	0	2.0	322.5	195.2
12	0.38	12.5	32.7	0	0	0	0	1.5	561.4	210.4
13	0.31	11.0	50.6	0	0	0	0	2.2	485.8	202.2
14	0.46	6.8	61.1	0	1.2	0	0	1.1	205.9	226.5
15	0.68	18.6	88.6	21.1	4.6	0	1.0	1.3	435.4	177.5
**Control**										
1	0.46	2.2	0	0	0	0	0	1.2	238.5	101.7
2	0	1.3	0	0	0	0	0	0.63	255.8	50.5
3	0.46	9.6	73.2	0	2.9	0	0	0.98	247.4	55.1
4	0.55	9.6	61.1	23.5	3.3	0	3.0	0.90	210.7	64.4
5	0.64	4.4	0	0	1.2	0	0	1.3	284.4	35.2
6	0.64	20.2	115.7	0	5.9	0	0	1.6	287.4	71.5
7	0	3.2	0	0	0	0	0	1.0	214.9	65.0
8	0	0.61	0	0	0	0	0	0	352.6	66.6
9	0.31	4.4	59.1	0	0	0	0	1.6	278.7	78.9
10	0.31	15.5	61.1	0	5.7	0	0	0.78	299.2	73.5

Interferon-gamma (IFN-γ), interleukin-2 (IL-2), interleukin-4 (IL-4), interleukin-6 (IL-6), interleukin-7 (IL-7), interleukin-8 (IL-8), interleukin-10 (IL-10), tumor necrosis factor-alpha (TNF-α), chemokine (CXC motif) ligand 1 (CXCL1), monocyte chemoattractant protein-1 (MCP-1).

**Figure 2:**
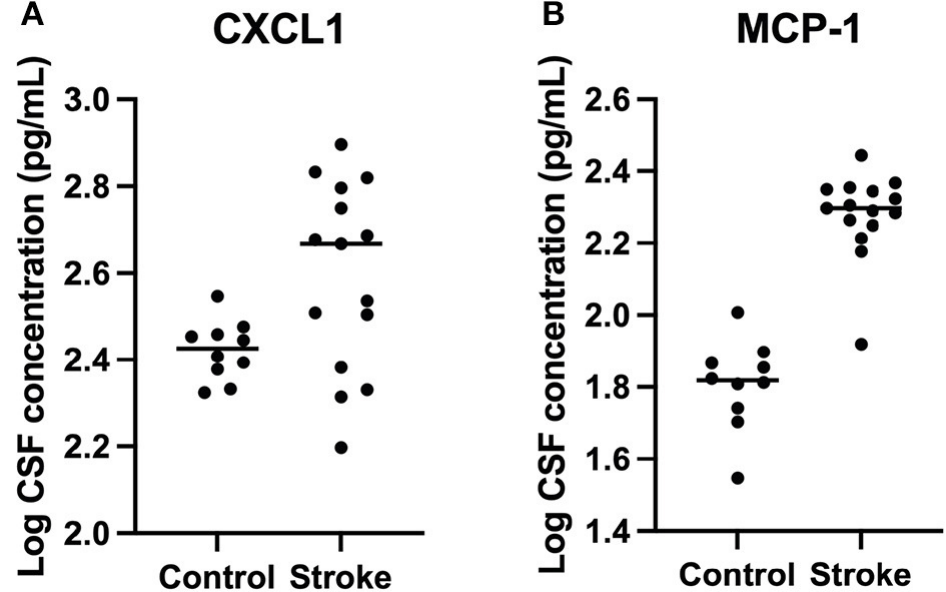
Logarithmically transformed cerebrospinal fluid CXCL1 and MCP-1 cytokine concentrations from dogs with stroke versus healthy controls. **(A)** CXCL1 (*p* = 0.01) and **(B)** MCP-1 (*p* < 0.0001) were significantly higher in stroke dogs compared with healthy controls.

CXCL1 and MCP-1 were detected in all cases and controls. Concentrations of CXCL1 were significantly elevated in dogs with stroke (mean = 436 pg/ml, media*n* = 466 pg/ml, range = 157–681 pg/ml) compared to control dogs (mean = 267 pg/ml, media*n* = 267 pg/ml, range = 210–352 pg/ml) (*p* = 0.01). Concentrations of MCP-1 were significantly elevated in dogs with stroke (mean = 196 pg/ml, media*n* = 198, range = 83–277 pg/ml) compared to control dogs (mean = 66 pg/ml, media*n* = 66 pg/ml, range = 35–101 pg/ml) (*p* < 0.0001) (Table 2 and Figure 2). Location and type of infarct, duration of clinical signs, and use of anti-inflammatory medications were not associated with differences in cytokine concentrations.

## 4. Discussion

Fifteen dogs with a diagnosis of ischemic stroke and 10 healthy control dogs had CSF evaluated for 14 cytokines: GM-CSF, IFN-γ, IL-2, IL-4, IL-6, IL-7, IL-8, IL-10, IL-15, IL-18, IP-10, CXCL1, MCP-1, and TNF-α. The majority of cytokines were detected in at least one case or control, and IFN-γ, IL-2, IL-4, TNF-α, CXCL1, and MCP-1 were detected in the majority of CSF samples analyzed. CXCL1 and MCP-1 concentrations were significantly elevated in cases of stroke compared to the controls.

This study expands on what has been previously reported by analyzing CSF for a large number of cytokines in dogs with naturally occurring stroke. The only previous report of cytokine measurement in CSF from dogs with naturally occurring stroke assessed for IL-2, IL-6, IL-8, and TNF. In concordance with other human and animal reports, that study reported elevations of IL-6 in CSF and plasma from dogs with stroke (4). IL-6 elevations were not identified in this study, possibly due to small case numbers or differences in study populations and assays used. Based on information from the manufacturer, the limit of detection for IL-6 in the previous study was 2.4 pg/ml, and the median concentration of IL-6 in dogs with stroke was 6.6 pg/ml (4). The limit of detection in the current study was 12.1 pg/ml. Future studies should use an assay with a lower limit of detection for IL-6 as this cytokine may be an important biomarker.

Elevation of CXCL1 and MCP-1 in CSF from dogs with stroke is consistent with data from other species. CXCL1 is a neutrophil chemoattractant that has been shown to be elevated in CSF from stroke patients, with higher levels being associated with larger stroke lesions on imaging, (8) and the blockage of CXCL1 receptors in mice protects against brain damage in models of ischemic stroke (9). MCP-1 is a chemokine known to recruit monocytes, T cells, and dendritic cells to sites of inflammation. MCP-1 is elevated in CSF from people with stroke, (10) and MCP-1-deficient mice have smaller infarct sizes in a middle cerebral artery model of stroke (11).

In addition to small case numbers, there are several other possible limitations to this study. Stroke cases were not confirmed by histopathological evaluation of brain tissue. Healthy controls were not age- and breed-matched, which may have altered cytokine concentrations. Additionally, since the stroke cases represent a true clinical population, there is a large degree of variability among cases, including breed, age, sex, and duration of clinical signs before CSF collection.

There are numerous considerations for the future study of cytokines in dogs with stroke. Prospective studies should aim to include larger case numbers, which will likely require multi-institutional involvement. Controls should be age-, breed-, and sex-matched. Findings could be correlated with the severity of clinical disease, and additional inclusion of volumetric MRI measurements would allow for the correlation of size of stroke with cytokine elevations. The inclusion of dogs with non-stroke CNS disease (e.g., inflammation, neoplasia) may also prove valuable since elevation of CSF cytokines is not specific to stroke. In people, elevated pro-inflammatory CSF cytokines have been identified in numerous CNS diseases, including multiple sclerosis and infectious encephalopathies (12, 13). In dogs, pro-inflammatory CSF cytokines have been identified after seizures, (14) in association with intervertebral disc disease, (15) and in association with neuropathic pain (16).

Although serum cytokines were not measured here, this should be considered for future studies as alterations of cytokine levels in peripheral blood could provide readily accessible biomarkers that may be used to predict the prognosis and risk of recurrence or to guide treatment (17). Elevations of numerous pro-inflammatory cytokines, including CXCL1 and MCP-1, have been identified in the serum of human stroke patients (18–20). These changes can be long-lasting, with IL-6 and TNF-α elevated in serum from people with stroke for at least 90 days (21). Furthermore, elevations in serum cytokines have been related to stroke severity, short-term prognosis, and infarct volume in people (18).

Although the results presented here must be validated in a larger clinical trial, the fact that MCP-1 is elevated compared to controls in all but one case suggests the timing of CSF collection may not be critical for this inflammatory chemokine, making it an enticing prospect for future translational research.

## Data availability statement

The raw data supporting the conclusions of this article will be made available by the authors, without undue reservation.

## Ethics statement

The animal study was reviewed and approved by UGA IACUC. Written informed consent was obtained from the owners for the participation of their animals in this study.

## Author contributions

SP and LD were responsible for sample acquisition. RB and JB performed the cytokine analysis. All authors were responsible for the study design, manuscript preparation, and approved the submitted version of the manuscript.
